# Laser Welding of 316L Austenitic Stainless Steel in an Air and a Water Environment

**DOI:** 10.3390/ma15062248

**Published:** 2022-03-18

**Authors:** Mohamad Alhajhamoud, Levent Candan, Mehmet Alp Ilgaz, Ibrahim Cinar, Sayit Ozbey, Selma Čorović, Damijan Miljavec, Ersin Kayahan

**Affiliations:** 1 Biomedical Engineering, Natural and Applied Sciences, Kocaeli University, Umuttepe, Kocaeli 41380, Turkey; mech24680@gmail.com; 2 Laser Technologies Research and Application Center (LATARUM), Kocaeli University, Yeniköy, Kocaeli 41275, Turkey; leventcandan@kocaeli.edu.tr (L.C.); ibrahim.cinar@kocaeli.edu.tr (I.C.); sayit.ozbey@kocaeli.edu.tr (S.O.); 3 Faculty of Electrical Engineering, University of Ljubljana, 1000 Ljubljana, Slovenia; selma.corovic@fe.uni-lj.si (S.Č.); damijan.miljavec@fe.uni-lj.si (D.M.)

**Keywords:** laser welding, austenitic stainless steel 316L, Nd: YAG laser, depth of penetration, microstructure

## Abstract

Laser welding is an innovative method that is frequently used and required by different disciplines and represents a technique of choice in a wide range of applications due to important advantages such as precision, speed, and flexibility. However, the welding method must be used properly otherwise it may deteriorate the mechanical properties of the welded metal and its environment. Therefore, the laser parameters should be precisely determined and carefully applied to the sample. The primary objective of this study was to investigate and propose optimal welding parameters that should be adjusted during the neodymium-doped yttrium aluminum garnet (Nd: YAG)-pulsed laser welding of austenitic stainless steel 316L in an air welding environment by using Argon shielding gas and in wet welding settings in serum medium. The investigation of the welding process in serum medium was conducted in order to propose the most suitable welding parameters being important for future possible medical applications of laser welding in in-vivo settings and thus to investigate the possibilities of the welding process inside the human body. In order to evaluate the quality of welding in air and of wet welding (in serum), a detailed parameter study has been conducted by variation of the laser energy, the welding speed and the focal position. The relationship between the depth of penetration and specific point energy (SPE) was also evaluated. The microstructure of the welded metal was examined by an optical microscope and scanning electron microscope (SEM). Based on the microscopy results, it was found that the largest depth of penetration (1380 µm) was achieved with 19 J laser energy in air medium, while the depth reached the largest value (1240 µm) in serum medium at 28 J laser energy. The increasing energy level showed opposite behavior for air and serum. The results of our study imply that when welding of 316L stainless steel is implemented properly in the body fluid, it would be a promising start for future in-vivo studies.

## 1. Introduction

Underwater welding finds its usage especially in the field of marine, nuclear power plant, offshore oil, and gas industries. By this welding technique, repairment and welding in deep-water areas have become applicable [1]. This method is classified into three main groups: dry welding, local cavity welding, and wet welding. Wet welding is more practical and has a lower cost compared to dry welding while it does not require any chamber for welding [2]. However, in the welding region, a large amount of hydrogen is absorbed and this causes crack formations in the microstructure. In dry welding, the amount of hydrogen is lower than that in wet welding, and the quality of the welding is strongly similar to atmospheric welding [3]. Underwater laser beam welding (ULBW) can promote phase transformation from ferrite to austenite. The refined microstructure can be obtained after the welding process. A decrease in the speed of welding can increase the period of high temperature. Therefore, the gas in the molten pool can escape, and the porosity of the welding area decreases [4]. Wet welding is more complex compared to welding in the open air due to the relatively high pressure, hydrogen percentage of welding metal, and a higher rate of cooling [2]. On the other hand, several promising studies have been conducted to enhance the underwater welding quality such as the liquid shielding with glycerol, since the welding area is less porous and has smoother morphology with this method [5]. The temper bead welding method is another application to improve the underwater weldability, which can reduce the hardness of the welding area [6]. In situ local heat treatment is beneficial for weldability in the air environment; however, it is not sufficiently verified for underwater welding. Tomków and Janeczek showed that in situ heat treatment with additional stitches improves the quality of welding [7].

The welding parameters depend on the thermal cycle of welding, the hydrogen amount, the transformation of phase, and the microstructure distribution after the weld re-solidification. Additionally, the water has a significant effect on laser transmission and the behavior of molten pools, and it creates a poor welded surface morphology [8,9].

The invention of the semiconductor laser logarithmically accelerated the developments of underwater welding. The laser power has been increased (more than one kilowatt), and the size of the laser machine has reduced in time. The primary advantages of ULBW are simple usage, low cost, and not requiring divers [10].

Austenitic stainless steel has proper formation for heat treatment and it has no magnetic characteristic in theory. It is a frequently used material in the manufacture of surgical instruments with 18–20% chromium and 8–10% nickel [11,12]. In addition, this type of steel has higher corrosion resistance and advanced mechanical properties. Thus, it is used in many scientific and industrial fields such as the petrochemical and medical industries. Moreover, austenitic steel is widely used in bone surgeries due to its high anti-rust properties. The most commonly used bio equipment is made of 316L stainless steel, and the letter “L” indicates the carbon content is low [13].

Austenitic stainless steel is appropriate for welding, and it prevents the formation of cracks and rust after the welding process. Because of those aspects, austenitic stainless steel is preferred for various purposes in chemical industries [14]. Underwater weldability of steel is an advantage that is strongly related to hot or cold cracking tendency [2]. Austenitic stainless steel can withstand contractions adequately because it is ductile enough. Besides, the welding area might crack in the cooling process [15]. One of the most common welding challenges for austenitic stainless steels is the precipitation of carbides since their microstructure is unbalanced at temperatures between 873 K–1173 K, where chrome joins the carbide structure [16].

The welding joint is generally evaluated by its chemical composition, mechanical advantages, and hardness compared to the base metal. In addition, it has high resistance to cracking at low temperatures. It also does not require heat treatment after welding due to the presence of a stable austenitic microscopic structure.

Laser welding is one of the useful techniques that provides high energy and fewer heat deformation and heat-affected zones (HAZs) [17]. After the welding process is completed, the dendrite arm spacing (DAS) and secondary dendritic arm spacing (SDAS) may form in the material, affecting the fatigue life period prediction. Besides, the fatigue life period is more vulnerable to SDAS scattering, especially in the case of small defects [18].

For the welding process, it is important to carefully select the appropriate welding speed and laser parameters [19,20]. The final mechanical properties of the welded samples are based on two different groups of parameters. The first group is related to the welding environment and welding period. The second group is related to the welding technique [21].

Austenitic stainless steel 316L is widely used in laser welding. However, austenitic stainless steel 316L undergoes hot cracking during welding as a result of liquation cracking and/or solidification cracking. Although several solutions have been proposed with the aim to avoid the hot-cracking, this effect still remains an important challenge to be addressed. It has been reported that sulfur, phosphorus, boron, silicon, and niobium negatively affect the welding quality of austenitic stainless steels. The drawbacks related to the welding of austenitic stainless steels are documented in [22].

Since laser welding in the underwater environment is becoming more and more popular in a wide range of applications, it is gaining more attention in numerous scientific investigations [1,23,24,25,26,27]. The novel applications of advanced wet laser welding include the laser methods applied in medical fields, which require a precise investigation of all physical parameters influencing the sample to be welded and its environment (i.e., medium). In this study, serum is used as an underwater welding liquid to predict the capability of welding on the human body being important for future in-vivo studies. The primary objective of this study was to investigate the key parameters of crucial importance for an efficient laser-welding procedure in two different media: underwater (i.e., serum medium) and atmospheric air. Therefore, the experimental measurements have been performed in order to investigate and compare the welding performance in the serum with atmospheric air welding using the Nd:YAG laser [made in Warwickshire, England, United Kingdom] for stainless steel 316L samples. The welding performance in both media was studied by the experimental investigation of the following key parameters: the laser energy, the welding speed, and the focal position. The welded samples were monitored by an optical microscope (OM) [made in Hamburg, Germany] and a scanning electron microscope (SEM) [made in Eindhoven, the Netherlands]. The formation of the microstructures of the stainless steel 316L samples was also investigated after the welding. Based on the microstructure analysis, the hot-cracking effect was investigated and the solution for its reduction proposed. Additionally, the specific point energy (SPE), which includes the parameters of the welding speed, the focal position, and the laser power, was evaluated to find the optimal welding parameters. It should be noted that the welding parameters for the wet laser welding we performed in the serum medium were examined in order to propose a novel welding method for a possible future in vivo laser welding of thin biomaterials. To the best of our knowledge, such an experimental investigation has not been performed previously and represents the novelty of the present study.

## 2. Materials and Experimental Study

The experimental study was performed on 316L stainless steel samples with dimensions 120 mm × 60 mm × 3 mm. Firstly, the samples were cleaned by an ultrasonic bath in acetone solution. Then, the laser welding was applied on the samples. After the welding process was completed, a water jet was used to cut small metal samples. The samples were metallurgically polished for the next step. In the process, the Nd: YAG laser that has a wavelength of 1064 nm with a millisecond pulse, was used due to the proper energy absorbance for 316L stainless steel. In this research, the laser power, the welding speed, and the focal position were selected as welding parameters. The influence of the parameters was analyzed by considering the heat-affected zone (HAZ) to find the optimum welding parameters. All welding processes were performed under laboratory conditions.

The chemical compositions and mechanical properties of 316L stainless steel are given in Table 1, respectively. The experimental parameters for the welding process are given in Table 2. Experimental parameters were specified according to optimum parameters of pre-experimental studies.

**Figure 1:**
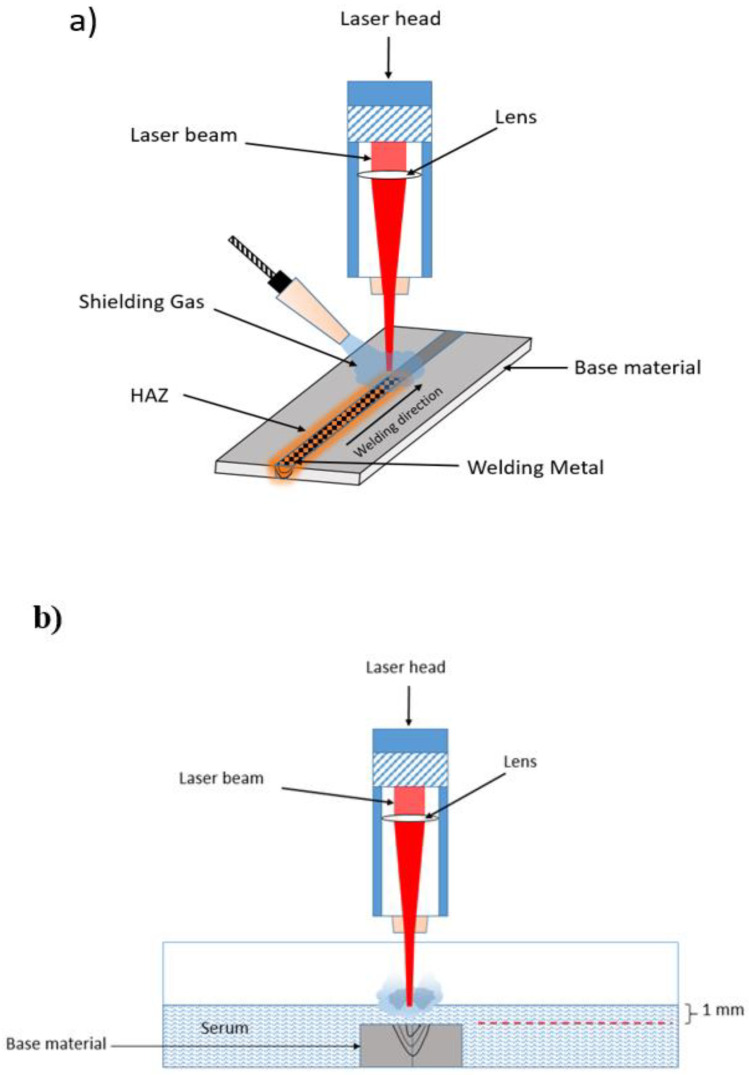
The schematic illustration of laser welding (**a**) in air and (**b**) in serum.

The welding process was performed in two different media: atmospheric air and serum. To avoid oxidation and improve joint performance, argon gas (18 L/min) was applied as protective gas for laser welding of stainless steel 316L [17,29]. For the underwater welding, serum liquid (which consisted of 0.9% salt, 0.154 mole Na and 0.154 mole Cl per liter, and 99.1% water) was used. The serums were purchased from the Biofleks OSEL Company in Turkey [Beykoz/Istanbul, Turkey]. The selected serum was preferred in order to facilitate the investigation of the laser welding quality in such a medium and thus to explore the possibility of the usage in medical applications. The schematic illustration of the welding process is given in Figure 1.

The *SPE* was calculated via a formula including the welding speed, energy, and focal position parameters as shown in Equation (1) [30]. The *SPE* was used to determine the influence of individual parameters. The principle of *SPE* is applied in laser welding since SPE can be used to assess the penetration range [30]. The *SPE* was calculated by the following equation:

(1) $$Specific\;Point\;Energy\;\left(SPE\right)\left(J\right)=P\frac{D}{V}$$

where *P* is the laser power [W], *D* is the laser beam diameter [mm], and *V* is the welding speed [mm/s]. The *SPE* calculation can be used to estimate the welding abilities for different laser parameters and systems [31].

To examine the microstructure with a good quality, the samples were etched by aqua regia (hydrochloric acid (*HCl*) 98 mL + nitric acid ($HNO_{3}$) 20 mL + glycerol ($C_{3}H_{8}O_{3}$) 60 mL). The microstructure was investigated by the image analysis software of the optical microscope Olympus BX51M [made in Olympus Life and Material Science Europa GBMH, Hamburg, Germany]

## 3. Results and Discussion

**Figure 2:**
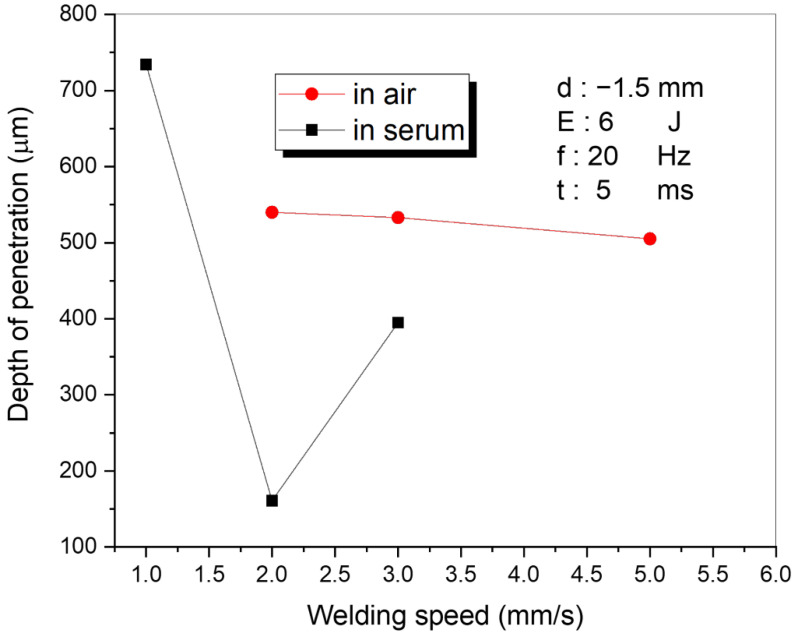
The relationship between the welding speed and the penetration depth.

The laser-welding process of the stainless steel 316L was performed in two different media (serum and air) in order to compare the welding performance and to find the optimum laser parameters. The most important parameters for evaluation of the laser-welding performance (i.e., well-known key factors for laser welding reported in the literature) such as the welding speed, the energy density, and the focal position were analyzed in both media. Within the first part of the study, the welding speed (mm/s) was varied while other parameters were kept constant (d = −1.5 mm, E = 6 J, f = 20 Hz, t = 5 ms). The welding variables are given in Table 2. The variations of the penetration depth with welding speed in both media are shown in Figure 2. Based on the results shown in Figure 2, it can be observed that the penetration depth slightly differed in the air; however, more visible differences could be observed in the serum medium. The laser power had a minor influence in the air when a low laser energy was applied (6 J). In the serum medium (when the welding speed increased from 1 mm/s to 2 mm/s), the depth of penetration decreased from 734 µm to 161 µm and increased from 161 µm to 395 µm for the speeds 2 mm/s to 3 mm/s. It is well-known that the penetration depth is mainly influenced by the laser power, the welding speed, and the beam diameter. In order to achieve a higher penetration depth during the laser welding, several solutions have been introduced/reported in the literature. For instance, a higher penetration depth can be achieved by increasing the laser power (and/or laser power to welding speed ratio), by decreasing the laser diameter or by pre-heating the weldment [32]. The abrupt change observed in Figure 2 may be due to the vaporization of water in the serum environment, which attenuates the amount of laser energy reaching the welding area. In addition, it may occur due to the fluctuations on the serum surface caused by the shock waves arising from the laser source. Conversely, in the air-welding environment, the penetration and the welding speed graph demonstrated a stable trend (i.e., almost constant).

**Figure 3:**
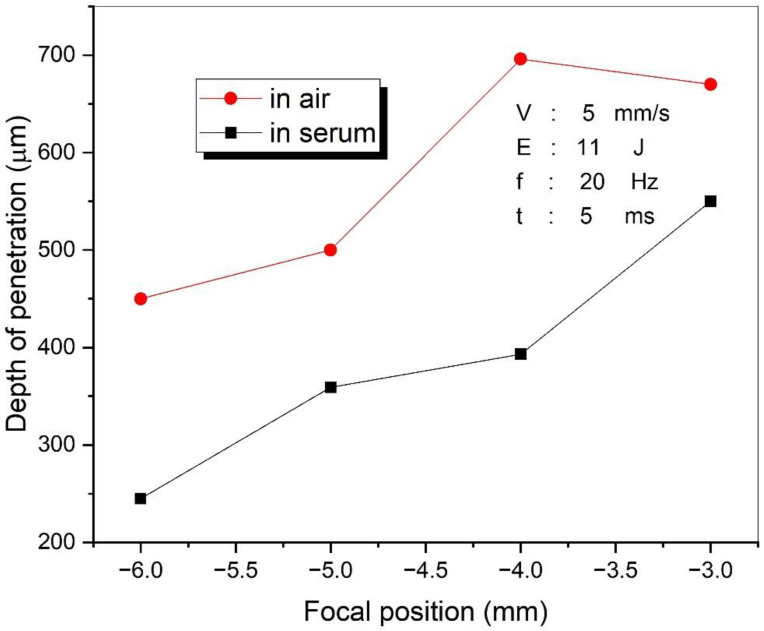
The variations in the penetration depth with the focal position.

The focal position of the laser beam may affect the laser-welding performance. The laser-welding process was performed with four different focal positions while other parameters were kept constant: 5 mm/s speed, 11 J energy, 20 Hz frequency, and 5 ms pulse duration. The change in penetration depth with various focal positions of the laser is shown in Figure 3. The penetration depth decreased significantly when the focal point distance increased from the welding surface, shown in Figure 3. When the focal point was far from the surface, the spot size of the laser increased as seen in Figure 3, which means the number of photons per unit area was lower. In addition, the penetration depth in air welding was greater than that in serum welding. This occurred because the serum medium absorbed more laser energy than the air medium.

**Figure 4:**
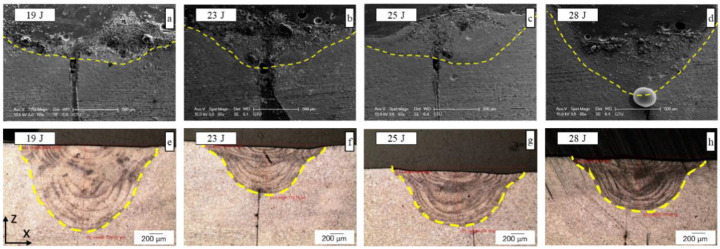
The relationship between the laser energy and penetration depth where focal distance d = −6 mm, welding speed V = 5 mm/s, frequency f = 20 Hz, and pulse duration t = 5 ms in serum obtained by SEM (**a**–**d**) and in air obtained by an optical microscope (**e**–**h**).

**Figure 5:**
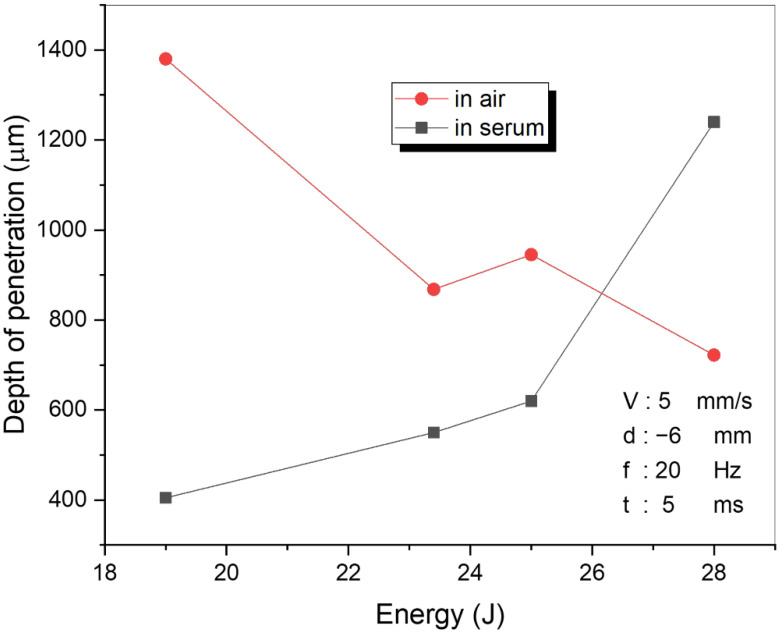
The relationship between the penetration depth and the laser energy.

The influence of the laser energy on the penetration depth and microstructure of the welded sample was also investigated. OM images and penetration depth values are given in Figure 4 and Figure 5, respectively. The results clearly showed that the laser energy had a solid impact on the penetration depth and microstructure. A high penetration depth of the laser-welding area was obtained with the 19 J laser energy in the air, compared with 28 J in the serum medium. In the air, after the laser energy exceeded 19 J, the penetration depth dramatically decreased as shown in Figure 4. In practice, the increasing laser energy caused increasing plasma in the welding region. The plasma within the region can be considered helpful, as it supports the join or absorption of the power in the laser beam by the weldments. Conversely, the plasma can jet out of the top of the welding region at higher laser powers (and/or slower welding speeds) and interact with the laser beam. The plasma produced by laser light absorbs the laser power and permits it to reach the weldment surface of the small part of the incident laser beam. Hence, this could be clearly explained by the laser–plasma interaction, where increased laser power decreases the penetration depth for laser welding in the air as seen in Figure 5. However, for the laser welding in the serum medium, the penetration depth increased with the laser energy as seen in Figure 5. The plasma created by the laser beam is suppressed by the liquid and stays in the keyhole, which also increases the penetration depth. In laser welding under liquid, the liquid height over the welding surface was selected carefully. Although the laser beam caused a beam channel in the liquid by shock waves, the laser beams were absorbed before they reached the surface at a very thick liquid height.

**Figure 6:**
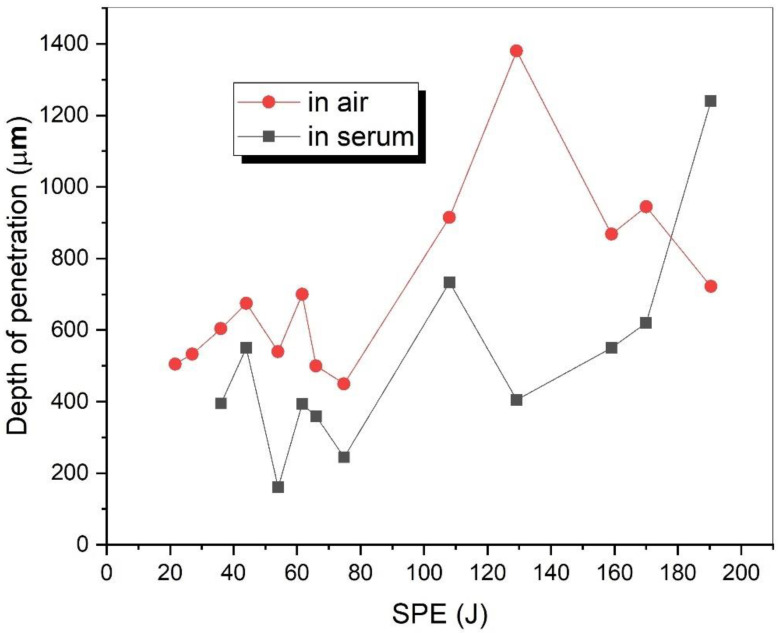
The relationship between the penetration depth and *SPE*.

Figure 6 shows the relationship between the *SPE* and penetration depth. In the air, the highest penetration depth (1380 μm) was obtained when *SPE* = 129.2 J. After this point, the penetration depth did not exceed the largest value, but it continued to fluctuate as shown in Figure 6. When the *SPE* increased from 21.6 J to 74.8 J, the penetration depth slightly changed due to heat conduction in the welding area. The jump in penetration depth from 450 μm to 1380 μm can be a reason for keyhole formation during welding at the *SPE* of 129.2 J [30]. The decrease in the penetration depth after this point can be explained by plasma formation due to high laser energy. In serum medium, the greatest penetration depth (1240 μm) was obtained at *SPE* = 190.4 J. Until *SPE* = 129.2 J, both medium results followed the same fluctuation path. After this point, the penetration depth increased significantly in serum while it decreased dramatically in the air. However, the results in Figure 6 do not show any visible correlation between the depth of penetration and the *SPE*. The results in Figure 6 show that the depth of penetration increased with the higher *SPE* values in the serum medium. However, the profile of penetration depth with respect to the *SPE* values in the air medium was more dynamic up to 75 J. After that value, the depth of penetration tended to increase (Figure 6).

In Ref. [7], the authors conducted an extensive experimental study focused on underwater laser welding by using high-strength low-alloy (HSLA) S355J2C + N steel. In their study, it was observed that the water environment caused some imperfections such as cracking and pores on the welded material surface. However, in our study, such deformations in the 316L stainless steel sample when the low welding energy was applied were not observed. This can be explained by the fact that the ions in the serum may prevent these phenomena. Therefore, this can be considered an important advantage of laser welding in serum environments.

## 4. Characterization of the Microstructure

**Figure 7:**
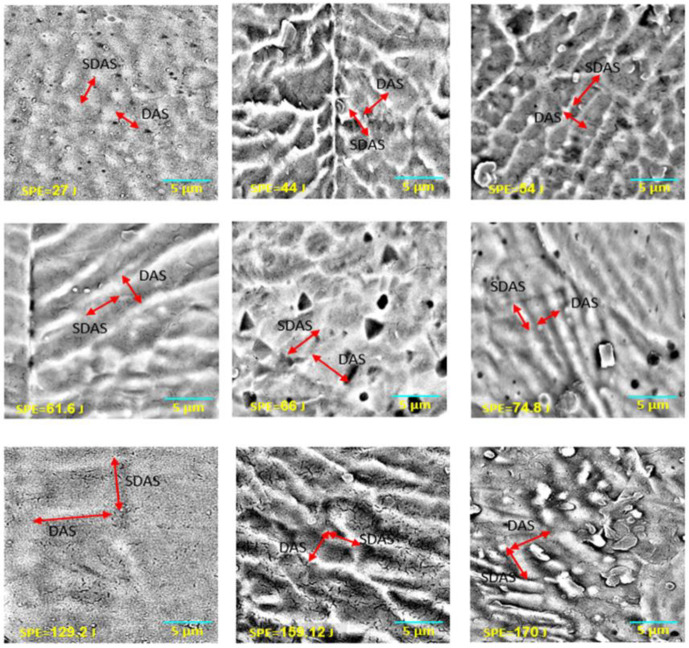
SEM images of the cross-section view of the 316L welded in air.

**Figure 8:**
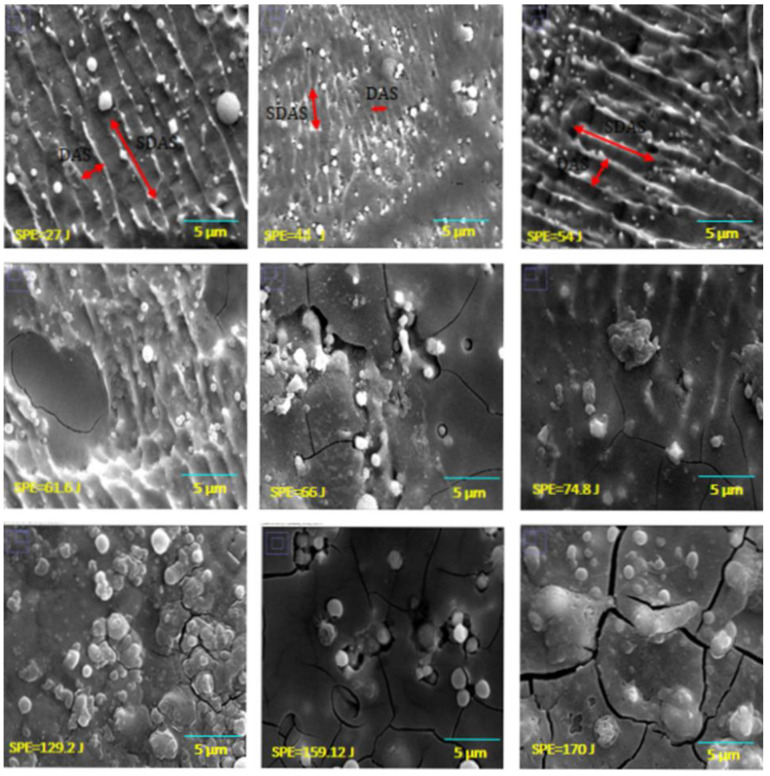
SEM images of the cross-section view of the 316L welded in serum.

**Figure 9:**
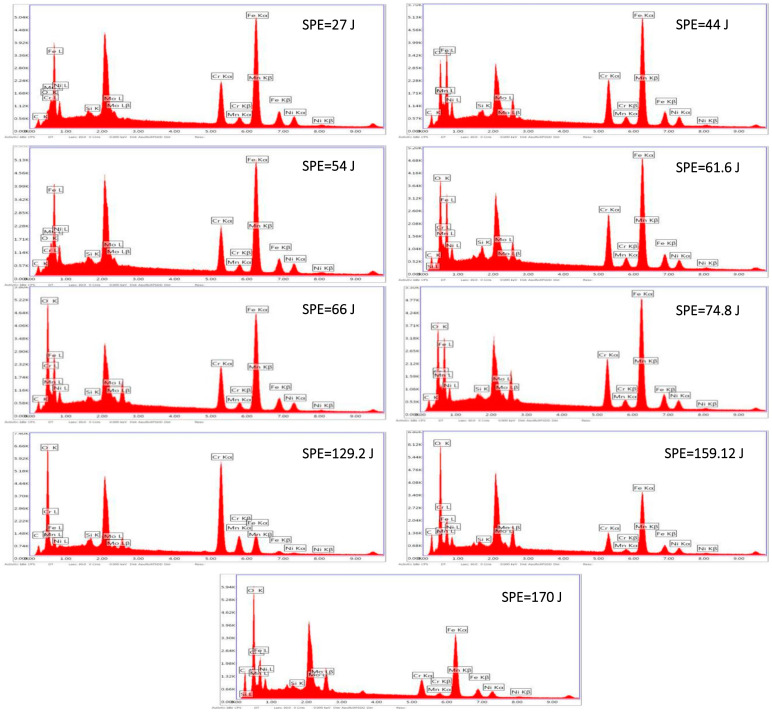
EDS images of the 316L weldment in serum medium.

As a result of applying a high concentration of laser energy, the temperature of the welded region increased dramatically, and the material in the welded area melted. This heat flow increased the temperature and affected not only the microstructure formation but also changed the hardness of the material in the welded area and the HAZ. Energy transfer to the joints created a melting pool. After the energy transfer process was completed, the melted material eventually solidified due to the rapid transfer of the heat. However, the structure did not return its original microstructure after the process. The structure of the treated sample changed, which is related to the redistribution of atoms based on the thermodynamic law [33]. Figure 7 and Figure 8 show the SEM images of the welded metal with various *SPE* values in air and serum media, while Figure 9 shows the EDS images of the laser welding samples in the serum medium.

It is clearly shown in Figure 7 that the DAS and SDAS structures were observed for all energy levels of the welding in the air medium. However, as seen in Figure 8, in serum medium welding, DAS and SDAS formed mostly at a lower *SPE* level (at 27 J, 54 J, 61.6 J) because of rapid solidification. The DAS values for serum medium welding at 27 J, 54 J, and 61.6 J were 1.27 µm, 1.02 µm, and 0.96 µm, respectively. The SDAS values for the same energy values were 4.03 µm, 4.74 µm, and 2.6 µm, respectively. Moreover, as seen in Figure 8, fracture formation occurred at the highest *SPE* value. This can be explained as follows: the shock waves that appeared due to laser light in the serum medium at high energy levels affected the welded surface. Mechanical properties are related to the dimensions of the DAS and SDAS. The smallest SDAS had the best properties of the materials [34]. During the process of serum welding, H_2_O solvated to hydrogen and oxygen molecules. Hence, the white points on the SEM images in Figure 8 represent the amount of oxygen. Figure 9 indicates the oxidation of samples under the laser welding in the serum medium. It is seen that the oxygen-related peak increased with the laser energy. This also proves that changes in the surface properties of the fusion zone were due to oxidation. In addition, cracks appeared due to the increase in oxidation. Cracking was not observed in the fusion zone when a low laser energy level was applied. This indicates that the solidification trains did not exceed the ductility of the solidifying weld metal as determined in [35].

One of the main drawbacks of the welding process of the austenitic stainless steel material is the hot cracking that is observed after the laser welding. Hot cracking is considered one of the main challenges to be addressed, as reported by numerous studies. In Ref. [36], the authors demonstrated via the Schaeffler diagram that fully martensite or full austenitic microstructure formation was responsive to the hot cracking. In addition, the authors also suggested that the austenitic microstructure with a 5~10% ferrite content was a desirable microstructure preventing hot cracking formation. In order to develop an appropriate solution to prevent hot cracking, several approaches have been proposed; however, this issue still remain a challenge. For instance, the fusion zone composition should be changed by referencing the Schaeffler diagram [36]. In addition, the authors suggested increasing the percentage of chrome (Cr) while decreasing the percentage of nickel (Ni) in the welded region.

**Figure 10:**
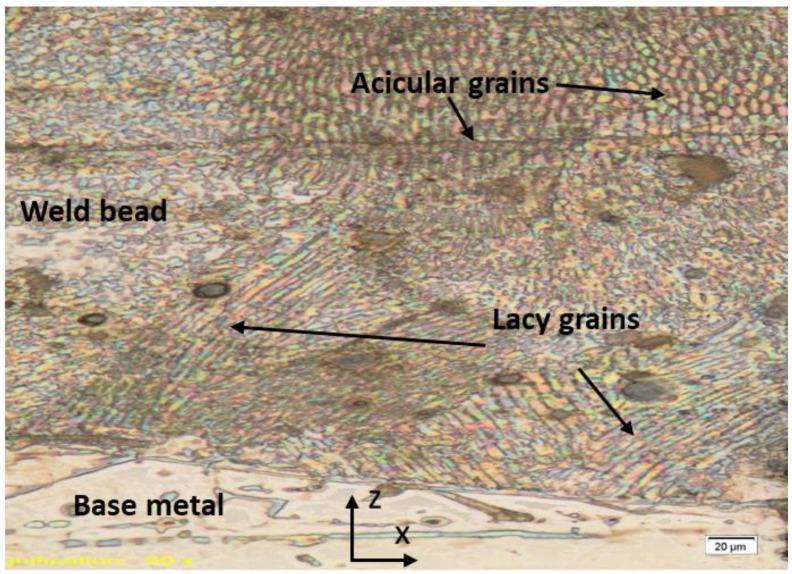
The microstructural view of the welding region in the serum medium.

When performing laser welding in the serum environment, an oxygen-enriched region was formed on the top of the welded sample, which agrees with the findings reported in Ref. [25]. The solidification of the welded region in the water environment occurred more rapidly than in the air environment due to the water-cooling effect. As a result of the described effect, a mixed crystal area with many grain boundaries was formed, as shown in Figure 10. A cross-section optic microscope image of the welded region is also given in Figure 10. The fusion zone contained an austenite phase consisting of grains with lacy and acicular forms due to high cooling rates as reported in Ref. [37]. The lacy form of ferrite was characterized by long columns of an interlaced ferrite network oriented along the growth direction in an austenite matrix. The ferrite was located within the cellular dendrites. The lacy form of ferrite occurred in the austenite matrix since the ferrite is characterized by the growth direction in an austenite matrix [37]. The ferrite structure could be seen in the cellular dendrites [37]. Based on similar findings that have already been reported in Ref. [37], the acicular morphology may be formed by random distribution of the needle-like ferrite in the austenite matrix.

## 5. Conclusions

In this study, an investigation of the laser welding of austenitic stainless steel 316L has been performed in two different environments, the air and a serum medium. The effects of key experimental parameters on the microstructure and the penetration depth of the austenitic stainless steel 316L samples were investigated. Based on the obtained results, the following conclusions have been derived:

- The welding speed has a greater influence on the penetration depth in the serum medium than in the air medium. The relationship between the welding speed and the penetration depth observed in our study demonstrates that the penetration depth in the air medium is almost stable with respect to the welding speed. However, the penetration depth in the serum medium decreases when the welding speed increases.
- With a smaller focal diameter of the laser, the depth of penetration increases in both media.
- The penetration depth is inversely related to the laser power in the air medium due to the laser-plasma interaction. Conversely, the penetration depth in the serum medium increases with the laser power.
- The experimental results show that there is no direct correlation between the *SPE* and the depth of penetration in both media.
- Our results demonstrate that oxidation occurs in the sample when laser welding is applied in the serum medium. In addition, the oxidation is more pronounced with higher *SPE* values. The EDS results also prove that the oxygen amount increases with *SPE*. The oxidation results in cracking in the fusion region. Thus, we suggest a lower *SPE* in order to prevent cracking in the fusion region.
- The austenite phase of the lacy and acicular grain structures has been observed in the fusion zone.
- In order to avoid the hot cracking formation, we also propose heating up the welded material by various heat sources during underwater laser welding, which will prevent the rapid cooling effect of the fusion zone (i.e., decayed cooling process). This means that the decayed cooling effect will result in the desired microstructure formation (i.e., the austenitic microstructure with 5~10% ferrite as previously mentioned) and therefore hot cracking will be avoided.

In conclusion, if the laser welding method of 316L stainless steel is implemented properly in the body fluid, it would be a promising start for future in-vivo studies. Therefore, the experimental results we present in this study indicate the that parameters significantly influence the performance of the conduction mode of laser welding. The results presented here can be used for development of a novel method for laser welding of thin biomaterials for which the experimental parameters (the laser power, the welding speed, the beam diameter) should be carefully selected in order to find the optimum depth of penetration as well as to prevent hot cracking. However, this technique requires further development and scientific investigation before the implementation of welding in the human body. Thus, the future work of this study will be focused on the laser-welding performance investigation in different biological media.

## Figures and Tables

**Table 1:** Chemical composition of the 316L stainless steel (Wt.%) [17]. Mechanical properties of the 316L stainless steel [28].

Chemical Composition (wt.%)									
**Sample**	**C**	**S**	**P**	**Si**	**Mn**	**Ni**	**Cr**	**Mo**	**Fe**
316L	≤0.03	≤0.03	≤0.045	≤1.0	≤2.0	10–14	16–18	2.0–3.0	Bal
**Mechanical Properties**									
**Tensile strength**		**Yield strength**			**Hardness**		**Elastic modulus**		
485 MPa		170 MPa			95 Rockwell		193 GPa		
**Physical Properties**									
**Density**		**Thermal conductivity**			**Specific heat 0–100 °C**		**Electrical resistivity**		
8000 kg/m^3^		16.3 at 100 °C		W/m·K	500 J/kg·K		740 nΩ·m		
		21.5 at 500 °C							

**Table 2:** Experimental parameters for the serum and air welding.

	Parameter	Symbol	Unit	Value
Variable	Energy density (heat input)	E/l	J/mm	60
				110
				190
				230
				250
				280
	Laser power	P	W	120
				220
				380
				460
				500
				560
	Welding speed	V	mm/s	2
				4
				5
	Focal position	d	mm	−1.5
				−3
				−4
				−5
				−6
Constant	Frequency	f	Hz	20
	Type of gas			Ar
	Gas flow (Argon)		L/min	18
	Pulse duration	t	ms	5

## Data Availability

Data sharing is not applicable for this paper.
